# Comparison of first-line and second-line terlipressin versus sole norepinephrine in fulminant ovine septic shock

**DOI:** 10.1038/s41598-018-25570-x

**Published:** 2018-05-08

**Authors:** Tim G. Kampmeier, Philip H. Arnemann, Michael Hessler, Laura M. Seidel, Karsten Becker, Andrea Morelli, Sebastian W. Rehberg, Christian Ertmer

**Affiliations:** 1 Department of Anaesthesiology, Intensive Care and Pain Medicine, University Hospital of Muenster, Muenster, Germany; 2 Institute of Medical Microbiology, University Hospital of Muenster, Muenster, Germany; 3 Department of Anaesthesiology and Intensive Care, University of Rome, “La Sapienza”, Rome, Italy; 4 Department of Anaesthesiology, Intensive Care, Emergency and Pain Medicine, University Hospital of Greifswald, Greifswald, Germany

## Abstract

The Surviving Sepsis Guidelines suggest the use of vasopressin in case of catecholamine-refractory septic shock. Terlipressin (TP) as a V_1_-selective AVP analogue is a potential alternative, though data regarding the first-line administration in septic shock are scarce. The present study explored and compared the effects of first-line vs. second-line infusion of TP or sole norepinephrine regarding organ function, fluid and norepinephrine requirements and survival in fulminant ovine septic shock. Peritoneal sepsis was induced in 23 ewes after laparotomy and faecal withdrawal from the caecum. After onset of shock, causal and supportive sepsis therapy (antibiotics, peritoneal lavage, fluids and open-label norepinephrine) was performed in all animals. Concurrently, animals were randomized to receive 0.9% sodium chloride (control group) or TP (2 µg∙kg^−1^∙h^−1^, first-line group) after shock onset. In the second-line TP group, TP (2 µg∙kg^−1^∙h^−1^) was started once norepinephrine requirements exceeded 0.5 µg∙kg^−1^∙min^−1^. No significant differences were found between groups regarding survival, haemodynamics as well as fluid- and catecholamine-requirements. Kidney function and electron microscopic kidney injury were comparable between groups. In the present model of fulminant ovine septic shock, first-line TP infusion had no significant effect on fluid and norepinephrine requirements or organ dysfunction as compared to second-line TP infusion or placebo.

## Introduction

Patients with septic shock commonly require large doses of catecholamines to maintain a sufficient mean arterial pressure (MAP). According to the current sepsis guidelines, norepinephrine is the vasopressor of choice in the treatment of sepsis related vasodilation^1^. However, there is increasing evidence that high catecholamine doses may have detrimental effects and is associated with increased mortality^2,3^. Thus, alternative, non-adrenergic vasopressors are desirable as first- or second-line treatment of sepsis-associated vasodilation.

The current sepsis guidelines suggest the vasopressin receptor agonist arginine-vasopressin (AVP) as second-line treatment if MAP cannot be maintained by norepinephrine alone. The second indication for non-adrenergic vasopressors is to reduce to dose of norepinephrine needed. First-line AVP therapy however, is discouraged by the guidelines in fear of ischemic end-organ events. Additionally, the reluctant use of AVP in clinical settings might be based on low experience and the fear of clinicians regarding intestinal or digital ischemia as well as reduced global oxygen delivery and cardiac output^4,5^. In contrast, evidence suggests that rather sepsis itself is the reason for such complications, and the use of vasopressin analogues does not trigger ischemic events^6,7^. Notably, administration of AVP in septic patients has been proven safe as supplemental (The Vasopressin in Septic Shock (VASST)-Trial) as well as first-line therapy (Vasopressin vs Norepinephrine on Kidney Failure in Patients With Septic Shock (VANISH)- Trial)^8,9^.

Notably, AVP is not available in several countries. Instead, the vasopressin-receptor agonist terlipressin (TP) is commonly used. TP has a higher selectivity for the V_1a_-receptor than AVP, and has been demonstrated equally or more effective than AVP in experimental and small clinical trials^10–12^. A single centre randomized controlled trial by Svoboda and colleagues with 30 patients investigated the effects of terlipressin administration in catecholamine-resistant septic shock. The authors concluded that continuous terlipressin infusion was ineffective in reduction of catecholamine requirements and mortality if applied in the late phase of catecholamine-resistant septic shock^13^. On the other hand, the previous published TERLIVAP-trial, which compared the effects of first-line AVP versus first-line TP in septic shock patients described a reduction in catecholamine requirements and lower rates of new onset tachyarrhythmias within the TP group^14^. Moreover, experimental data suggest, that V_1_ agonists may reduce sepsis-associated endothelial injury and capillary leakage, thus favouring early treatment initiation^15,16^.

Notably, no studies have yet investigated first-line versus second-line treatment with TP as a continuous infusion in septic shock. Therefore, the present study was designed to explore the effects of first-line continuous low-dose administration of TP versus second-line administration (which is the common situation in the clinical setting) regarding fluid and norepinephrine requirements as well as organ function and survival in fulminant ovine septic shock.

## Material and Methods

### Animal care

After arrival in the research facility, the animals were housed in flocks of 3 to 10 animals under veterinary supervision. Veterinary care attendants visited the sheep twice a day and more often when necessary. A veterinary examination of health status took place on arrival, prior to inclusion in the study and whenever deemed necessary by the veterinary care attendants. All methods were performed in accordance with the National Institutes of Health Guide and as well as the American Physiologic Society’s “Guide for the Care and Use of Laboratory Animals” using established protocols.

### Instrumentation

After approval by the Animal Care Committee of the State Government of North-Rhine Westphalia (LANUV NRW, Recklinghausen, Germany) with the approval (ref no. 8.87–50.10.37.09.194), 23 healthy female sheep (median body weight 42.0 kg, 34.0–43.5; 25^th^− 75^th^ percentile) were anaesthetized by intramuscular injection of S-ketamine (Ketanest^®^ S, 10 mg·kg^−1^, Parke-Davis, Berlin, Freiburg, Germany) and midazolam (Dormicum^®^, 0.3 mg·kg^−1^, Hoffmann-La Roche AG, Grenzach-Wyhlen, Germany). The ewes were held in abstinence from food for 12 hours prior to the instrumentation with free access to water. After endotracheal intubation with a 9.0 tracheal tube (Rüsch, Rüschelit^©^, Teleflex Medical GmbH, Kernen, Germany), anaesthesia was maintained by inhalational isoflurane with an expiratory fraction of 1.0–1.5% (Forene^®^; Abbott GmbH & Co. KG, Wiesbaden, Germany). A quadlumen central venous catheter (6 Fr. Quadlumen Catheter Set, PVB Medizintechnik GmbH, Kirchseeon, Germany) was placed using Seldinger’s technique into the right jugular vein through which anaesthesia was supplemented with S-ketamine (1 mg·kg^−1^·h^−1^), midazolam (0.3 mg·kg^−1^·h^−1^) and lidocain (1.5 mg·kg^−1^·h^−1^)^17^ during the further instrumentation. For continuous hemodynamic surveillance, a pulse contour cardiac output (PiCCO) catheter was placed in the right femoral artery (5 Fr.; Pulsion Medical Systems, Munich, Germany) with connection to a transpulmonary thermodilution and pulse contour cardiac output computer (PiCCO_2_, Pulsion Medical Systems, München, Germany). A Foley catheter (12 Fr. urinary catheter, Porgès S.A., Le Plessis Robinson-Cedex, France) was inserted to determine urinary output.

### Surgical preparation

Following a median laparotomy, the cecum of the animals was detected and incised in order to withdraw 1.5 g·kg^−1^ faeces. A contamination of the peritoneal cavity was strictly avoided. Two 16 Fr. drains were placed in the mesentery of the small intestine and the abdomen was closed with continuous suture afterwards. After a 2 hours’ phase of recovery, baseline (BL) data were assessed to examine whether the animals fulfil the inclusion criteria.

### Inclusion criteria

The following criteria had to be fulfilled at BL before inclusion in the study:

- Heart rate (HR) < 100 bpm
- Mean arterial pressure (MAP) 70–120 mmHg
- Cardiac index (CI) 2.5–6.0 L·min^−1^·m^2^
- Serum lactate ≤1,2 mmol·l^−1^
- Temperature 38.0–39.8 °C
- Arterial pH: 7.30–7.50
- Arterial carbon dioxide pressure 35–55 mmHg.

The inclusion criteria were based on reference values for healthy sheep^18^.

### Induction of septic shock

After inclusion in the study, autologous faeces were injected into the abdominal cavity via one of the 16 Fr. drain. Onset of septic shock was defined as

- MAP <60 mm Hg and
- Serum lactate concentration ≥1.8 mmol·l^−1^ (i.e. 1.5 times the upper normal limit of sheep^18^) and
- Minimum of four hours after instillation of the faeces.

After the onset of septic shock, “shock time” measurements were performed as detailed below.

### Randomization

After the “shock time” measurements, the animals were randomly assigned to one of the following study groups:

- Control (n = 7)

- [study solution 1: 0.9% saline; study solution 2: 0.9% saline]

- Terlipressin first-line (n = 8)

- [study solution 1: TP (2 µg∙kg^−1^∙h^−1^); study solution 2: 0.9% saline]

- Terlipressin second-line (n = 8)

- [study solution 1: 0.9% saline; study solution 2: TP (2 µg∙kg^−1^∙h^−1^)].

The attendant investigators were blinded for study group allocation and content of study drug syringes. Study solution 1 was started immediately after randomisation. The second study solution was initiated when norepinephrine requirements exceeded 0.5 µg∙kg^−1^∙min^−1^. Once initiated, both study solutions were administered with a fixed infusion rate until the end of the protocol.

### Study protocol

After randomization, study solution 1 was started as specified in the group description and continued throughout the whole experiment. Causal therapy was initiated by intravenous antimicrobial therapy with a bolus infusion of 20 mg·kg^−1^ meropenem (Meronem^©^, AstraZeneca GmbH, Wedel, Germany) and followed by continuous intravenous infusion with 2.5 mg·kg^−1^·h^−1^. Furthermore, peritoneal lavage was initiated by fractional instillation of four litres of warm (38° Celsius) saline through the abdominal drains until no more faecal contamination was detected.

Supportive fluid therapy was based on dynamic and volumetric hemodynamic parameters. Indications for fluid resuscitation were:

- Global enddiastolic volume index (GEDI) < 620 mL·m^−2^ or < BL1 value
- Stroke volume variation (SVV) > 13%
- Haematocrit (Hct) > BL1 value.

Contraindications for fluid resuscitation were:

- Extravascular lung water index (ELWI) ≥ 17 mL·kg^−1^
- Horowitz-Index (PaO_2_/FiO_2_) <2.

Fluid resuscitation was performed with hydroxyethyl starch (HES) 6% 130/0.4 (Volulyte^©^, Fresenius Kabi, Bad Homburg, Germany) and balanced crystalloids (Sterofundin^©^ ISO, B. Braun Melsungen, Germany). HES and crystalloids were applied alternately (250 ml HES followed by 500 ml crystalloid) until the maximum dose of HES was reached (50 mL·kg^−1^). If necessary, further fluid resuscitation was performed with crystalloids only until hemodynamic indicators were met.

Norepinephrine was initiated at the onset of shock and titrated to maintain a MAP ≥65 mmHg up to a maximum dose of 5 µg·kg^−1^·h^−1^. If norepinephrine requirements exceeded 0.5 µg∙kg^−1^∙min^−1^, the second study solution was initiated as detailed in the group description and continued until the end of the experiment. The maximum dose of norepinephrine was drawn from clinical experience, when no more vasoconstrictive effect of the substance could be expected due to tachyphylactic effects.

### Measurements

Hemodynamic parameters, urinary output as well as arterial and central-venous blood gas analyses were documented at BL, shock time and hourly thereafter. Blood and urine samples for laboratory and microbiological analyses were taken at BL, shock time and every four hours afterwards. The samples were immediately centrifuged and stored at −70 °C for later analyses.

### Analysed laboratory variables

The following variables were determined from blood and urine samples, respectively:

- Blood gas analyses (electrolytes, oxygen- and carbon dioxide partial pressure, pH, base excess (BE), haemoglobin, haematocrit, oxygen saturation, lactate, glucose).
- Parameters of organ (dys-) function (bilirubin, aspartate aminotransferase (ASAT), alanine aminotransferase (ALAT), serum creatinine concentration, serum urea concentration, creatinine-clearance).

Aerobic and anaerobic blood cultures were withdrawn under sterile conditions at BL, shock time as well as 8 h, 16 h and 24 h afterwards.

### End of protocol and autopsy

At the end of the 24 hours interventional period after shock time the animals were killed in deep propofol anaesthesia (4 mg·kg^−1^) with a bolus injection of 100 ml of 1-molar potassium chloride solution. All animals were autopsied with removal and weighing of the heart, lungs, kidneys and terminal ileum. Additionally, samples from the kidney were collected for electron microscopic analyses.

### Electron microscopy

Transmission electron microscopy (TEM) was performed with a Philips CmlO-Electronic microscope (Philips, Eindhoven, Netherlands) at 80 kV. Cellular damage, cellular oedema and mitochondrial damage was quantified by a pathologist who was blinded for the protocol. Ultrastructural kidney damage was quantified according to the “electron microscopic tubular injury” (EMTI) score^19^. This score contains the four criteria (1) vacuolar degeneration and swelling of organella, (2) dissociation of epithelium and basal membrane, (3) epithelial cell injury and (4) intratubular precipitation. Each criterion was scored from 0 to 3, thus the total EMTI score (sum of the four criteria) could range from 0 to 12 points^19^.

### Statistical analysis

Statistical analysis was performed with IBM SPSS statistics software version 22 (IBM, Armonk, New York, United States). All data are presented as median and interquartile range (IQR). Comparisons between groups for variables measured only once were made using Kruskal-Wallis H-test. If necessary, post-hoc comparisons were conducted using Dunn’s test. Comparisons between time points were made using Wilcoxon signed-rank test. Comparisons between groups for repeatedly measured variables were conducted by calculation of generalized estimating equations (GEE) with group as factor and time as covariate^20^. Asymptotic two-sided p-values smaller than 0.05 were assumed as statistically significant.

### Data availability

The datasets generated and analysed during the current study are available from the corresponding author on reasonable request.

## Results

### Features of septic shock (prior to study drug infusion)

All animals developed septic shock between BL and shock time with reductions in MAP, CI and with lactic acidosis (see Supplemental Digital Content 1 Table 1, BL versus shock time data). Renal function decreased during this time and acute kidney injury occurred, which was classified according to the KDIGO guidelines^21^ using diuresis and creatinine concentration (see Supplemental Digital Content 1 Table 1, BL versus shock time data).

**Table 1:** Haemodynamics of the study groups during the 24-hour interventional period.

Variable	Group	Shock time	4 h	8 h	16 h	24 h
CVP [mmHg]	Control	1 [0; 4]	7 [4; 8]	5 [2; 13]	11 [9; 17]	17 [12; 18]
	TP first-line	1 [0; 2]	4 [1; 9]	7 [2; 11]	5 [3; 9]	12 [7; 13]
	TP second-line	0 [0; 0]	2 [1; 4]	4 [2; 7]	8 [4; 10]	12 [6; 14]
EVLWI [mL·kg^-1^]	Control	13 [12; 17]	14 [11; 15]	14 [10; 15]	12 [11; 12]	12 [11; 17]
	TP first-line	14 [12; 21]	14 [12; 15]	15 [13; 18]	13 [12; 15]	12 [11; 13]
	TP second-line	13 [12; 16]	13 [12; 16]	14 [13; 19]	14 [11; 19]	11 [11; 13]
GEDI [mL·m^−2^]	Control	606 [494; 644]	760 [728; 848]	741 [642; 781]	631 [572; 695]	641 [616; 766]
	TP first-line	599 [528; 739]	791 [665; 847]	740 [668; 833]	691 [665; 788]	700 [603; 730]
	TP second-line	509 [409; 595]	725 [663; 801]	708 [683; 810]	686 [596; 720]	656 [598; 788]
MAP [mmHg]	Control	40 [36; 54]	60 [56; 61]	60 [58; 61]	63 [60; 64]	62 [61; 63]
	TP first-line	46 [35; 49]	60 [50; 62]	61 [59; 63]	64 [63; 64]	61 [60; 61]
	TP second-line	39 [34; 41]	58 [58; 61]	60 [56; 61]	63 [61; 65]	60 [54; 60]
SVI [mL·m^−2^]	Control	28 [24; 35]	57 [48; 70]	57 [44; 74]	48 [45; 66]	59 [47; 74]
	TP first-line	24 [19; 30]	39 [36; 52]	57 [41; 66]	45 [44; 50]	50 [38; 57]
	TP second-line	23 [21; 29]	50 [45; 58]	56 [48; 62]	33 [23; 56]	33 [23; 56]
SVRI [dyn·s/cm^−5^·m^−2^]	Control	1497 [1208; 1883]	630 [449; 1037]	612 [515; 1001]	727 [548; 926]	846 [458; 886]
	TP first-line	1352 [1276; 1912]	729 [513; 946]	680 [518; 1000]	993 [799; 1223]	741 [609; 1028]
	TP second-line	1509 [1434; 1647]	666 [617; 857]	603 [531; 718]	818 [638; 1037]	741 [559; 1405]
SVV [%]	Control	11 [8; 13]	12 [11; 15]	11 [9; 16]	12 [9; 16]	14 [10; 18]
	TP first-line	8 [5; 11]	10 [9; 16]	13 [12; 14]	12 [10; 12]	14 [12; 15]
	TP second-line	16 [11; 19]	12 [11; 14]	15 [11; 17]	11 [10; 14]	14 [10; 19]

Values are presented as median [interquartile range].

*CVP, central venous pressure; EVLWI, extravascular lung water index; GEDI, global end-diastolic index; SVI, stroke volume index; SVRI, systemic vascular resistance index; SVV, stroke volume variation; TP, Terlipressin*.

### Hemodynamic and oxygen transport variables (during study drug infusion)

There were no differences between the study groups regarding hemodynamic variables (see Table 1, Supplemental Digital Content 2 Fig. 1 and Supplemental Digital Content 3 Fig. 2, cardiac index and heart rate). Haematocrit was higher within the TP second-line group (p < 0.05) as compared to TP first-line group (see Supplemental Digital Content 4 Fig. 3, Haematocrit concentration). All other measured parameters of oxygen transport were comparable between the study groups (see Table 2).

**Figure 1:**
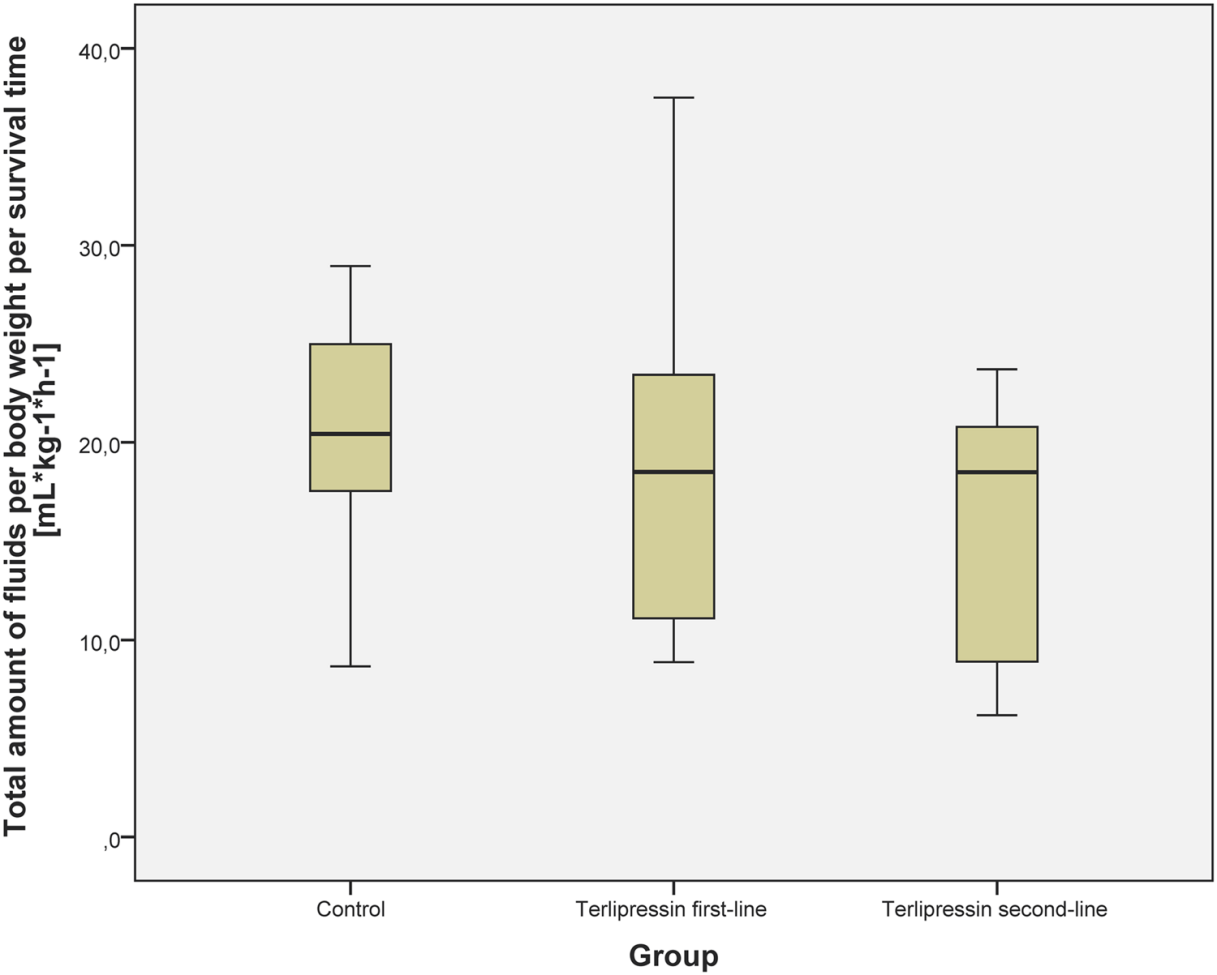
Cumulative fluid amount of the study animals. The figure demonstrates the cumulative fluid amounts of the study animals over the 24-hour interventional period. Data are presented as median [interquartile range].

**Figure 2:**
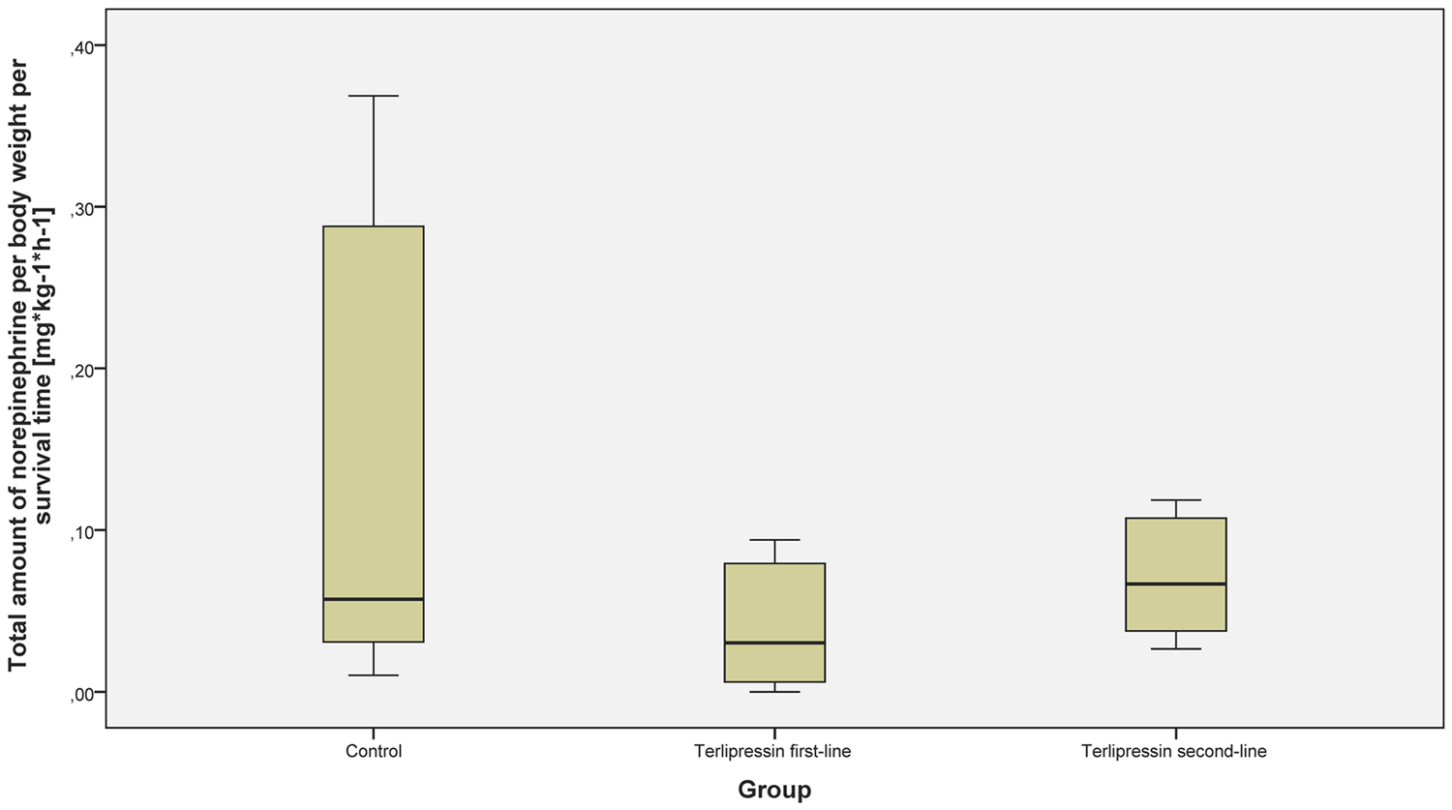
Cumulative norepinephrine amount of the study animals. The figure demonstrates the cumulative norepinephrine dose of the study animals averaged over the 24-hour interventional period. Data are presented as median [interquartile range].

**Figure 3:**
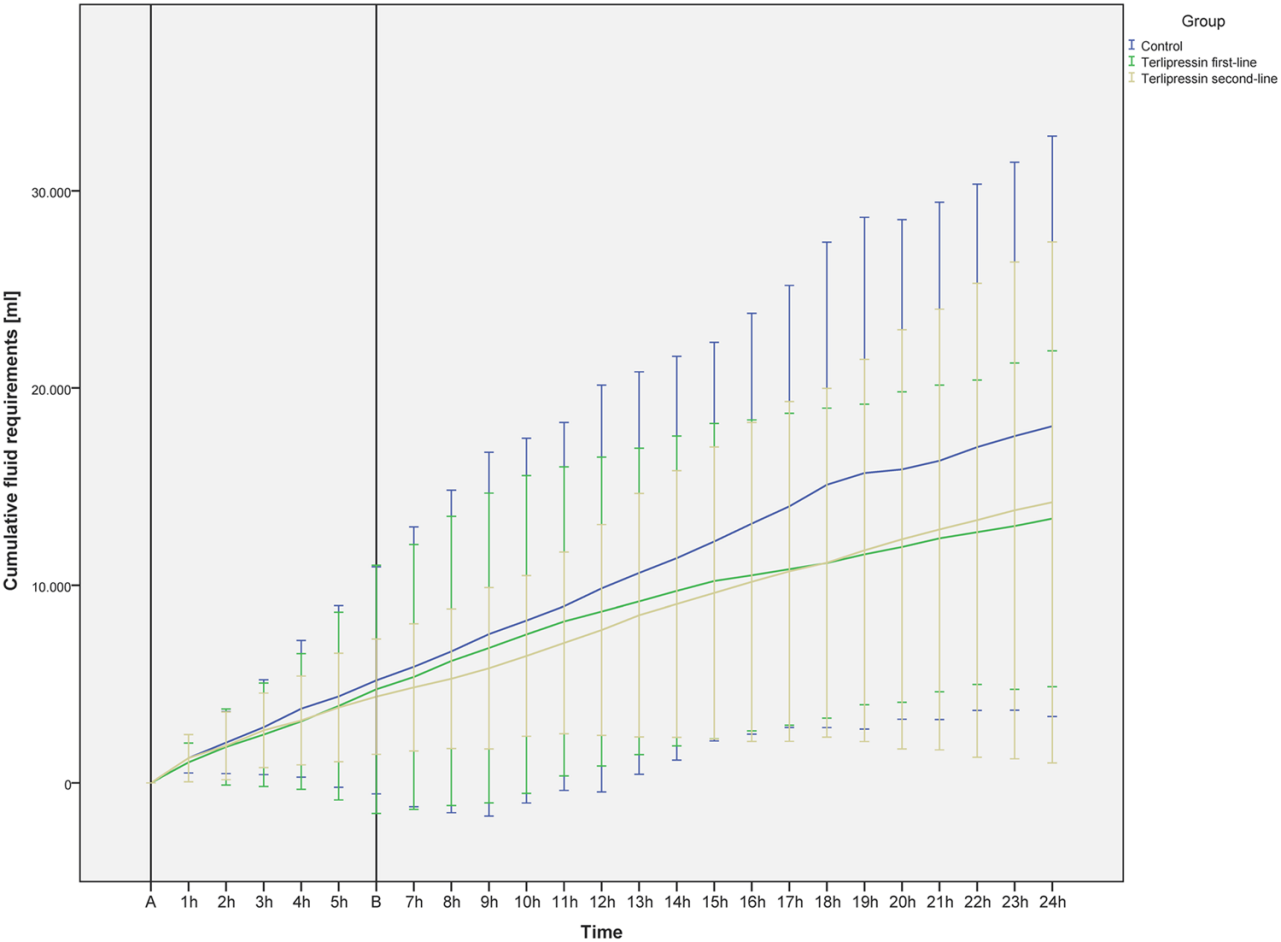
Cumulative fluid requirements per hour. The figure demonstrated the cumulative fluid requirements of the study animals over time within the 24-hour interventional period. The average initiation-points of the study solutions are highlighted in the figure. (A) Shock time and initiation of the 1^st^ study solution. (B) Average start of the 2^nd^ study solution. Data are presented as mean [standard deviation].

**Table 2:** Metabolism, oxygenation and temperature of the study groups during the 24-hour interventional period.

Variable	Group	Shock time	4 h	8 h	16 h	24 h
pH(a) [-lg c(H^+^)]	Control	7.44 [7.38; 7.48]	7.45 [7.43; 7.48]	7.42 [7.40; 7.45]	7.38 [7.16; 7.40]	7.22 [6.99; 7.27]
	TP first-line	7.39 [7.37; 7.44]	7.41 [7.35; 7.46]	7.42 [7.34; 7.45]	7.31 [7.23; 7.31]	7.20 [7.20; 7.24]
	TP second-line	7.45 [7.40; 7.49]	7.43 [7.40; 7.45]	7.39 [7.33; 7.44]	7.28 [7.10; 7.41]	7.10 [6.80; 7.31]
BE [mmol·L^−1^]	Control	7.0 [3.5; 8.5]	3.0 [2.2; 4.5]	2.2 [−0.3; 3.7]	−2,4 [−5.4; 1.7]	−5.6 [−7.2; −3.4]
	TP first-line	4.9 [2.1; 5.4]	1.0 [0.4; 1.9]	0.8 [−2.3; 1.9]	−3.3 [−3.6; −2.6]	−5.5 [−6.7; −5]
	TP second-line	6.6 [3.5; 7.4]	2.3 [1.4; 2.8]	1.4 [0.7; 3.2]	−3.2 [−7.1; 0.8]	−6.1 [−6.5; −4.9]
DO_2_I [mL·min^−1^·m^−2^]	Control	314 [251; 353]	608 [484; 727]	600 [456; 675]	467 [339; 642]	486 [348; 624]
	TP first-line	415 [387; 657]	585 [507; 605]	543 [376; 661]	473 [331; 588]	559 [369; 674]
	TP second-line	243 [210; 357]	563 [418; 642]	669 [497; 793]	565 [488; 695]	466 [399; 481]
VO_2_I [mL·min^−1^·m^−2^]	Control	118 [98; 130]	79 [74; 91]	80 [65; 107]	74 [52; 101]	66 [47; 79]
	TP first-line	167 [107; 221]	120 [93; 130]	86 [55; 116]	63 [43; 66]	58 [43; 73]
	TP second-line	123 [90; 138]	97 [70; 105]	76 [64; 107]	72 [59; 92]	48 [44; 62]
O_2_-ER [mL·min^−1^·m^−2^]	Control	40 [39; 45]	14 [12; 19]	15 [11; 22]	19 [13; 21]	17 [11; 19]
	TP first-line	37 [31; 42]	19 [14; 27]	14 [11; 20]	13 [10; 17]	11 [7; 17]
	TP second-line	48 [36; 52]	20 [14; 24]	14 [11; 16]	13 [12; 15]	16 [10; 18]
ScvO_2_ [%]	Control	61 [60; 65]	85 [82; 87]	84 [80; 87]	81 [80; 84]	80 [79; 86]
	TP first-line	69 [64; 73]	84 [77; 88]	85 [78; 90]	87 [81; 90]	85 [78; 90]
	TP second-line	53 [47; 64]	83 [78; 86]	88 [83; 90]	82 [78; 87]	69 [60; 84]
Temperature [°C]	Control	39.4 [39.2; 40.1]	39.3 [38.7; 39.9]	39.4 [39.1; 40.0]	39.2 [38.7; 39.4]	39.5 [38.5; 39.6]
	TP first-line	40.0 [39.8; 40.5]	39.6 [38.9; 40.6]	39.7 [38.4; 40.9]	39.7 [38.7; 41.2]	39.6 [39.0; 41.1]
	TP second-line	40.3 [39.9; 40.4]	39.2 [39.1; 39.6]	39.6 [39.3; 40.0]	39.9 [39.4; 40.1]	39.7 [39.2; 40.9]

Values are presented as median [interquartile range].

*BE, base excess; DO*_2_*I, oxygen delivery index; O*_2_*-ER, oxygen extraction rate; pH(a), arterial potentia hydrogenii; S*_*cv*_*O*_2_*, central venous oxygen saturation; TP, Terlipressin; VO*_2_*I, oxygen consumption index*.

### Fluid and norepinephrine requirements

There were no differences between the study groups regarding cumulative fluid and norepinephrine requirements (see Figs 1, 2 and 3) over the 24-hour interventional period, though the catecholamine-requirements in the TP first-line group tended to be lower without statistical significance (median norepinephrine requirements per body weight per hours alive [µg·kg^−1^·h^−1^]: control group 57.2 [30.9; 287.9]; TP first-line 30.3 [6.1; 79.3]; TP second-line 66.6 [37.7; 107.3]). The initiation of the second study solution was after 6.0 h [4.0; 11.5] in the control group, 5.0 h [2.0; 6.0] in the TP first-line group and 6.0 h [5.0; 6.0] in the TP second-line group. Mean start of the second study solution over all groups was 6.2 h [±4.1] after shock time. The cumulative fluid requirements of the TP groups were lower as compared to the control group without statistical significance (see Figs 1 and 3).

### Organ function and EMTI-Score

**Figure 4:**
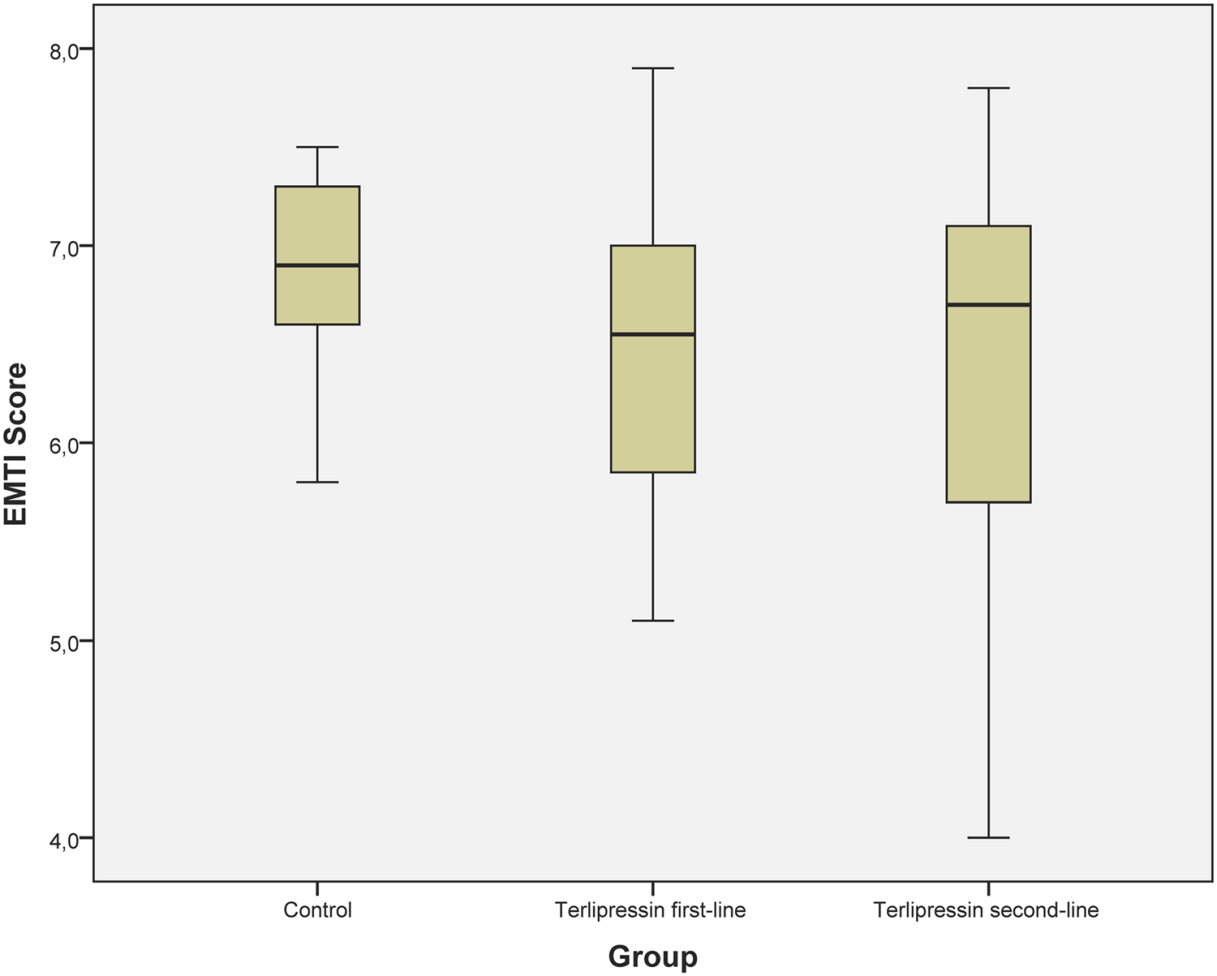
EMTI score of the study animals. The figure demonstrates the electronic microscopy injury (EMTI) score of the animals analysed in kidney biopsies. EMTI, electronic microscopy tubular injury. Data are presented as median [interquartile range].

All animals developed acute kidney injury at shock time, which persisted despite study therapy (see Table 3). Kidney function and injury were comparable between groups as measured by serum creatinine, creatinine clearance, diuresis and EMTI score (see Fig. 4 and Table 3).

**Table 3:** Parameters of organ function of the study groups during the 24-hour interventional period.

Variable	Group	Shock time	4 h	8 h	16 h	24 h
Lactate [mmol·L^−1^]	Control	1.8 [1.8; 1.9]	2.7 [2.4; 3.0]	2.9 [2.5; 3.2]	2.9 [2.7; 3.2]	2.2 [1.8; 3.8]
	TP first-line	1.9 [1.9; 2.0]	3.3 [3.1; 3.5]	3.6 [2.9; 3.9]	3.3 [2.3; 3.5]	2.6 [2.5; 3.2]
	TP second-line	1.8 [1.8; 2.2]	2.6 [2.2; 2.9]	2.6 [2.3; 3.5]	2.8 [2.1; 4.7]	3.3 [3.1; 3.6]
Creatinine [mmol·L^−1^]	Control	1.5 [1.2; 1.7]	1.1 [0.9; 1.2]	1.2 [1; 1.4]	1.1 [0.9; 1.7]	1.3 [1.1; 2.3]
	TP first-line	1.8 [1.3; 1.9]	1.4 [1.1; 1.8]	1.5 [1.1; 1.6]	2.2 [0.9; 2.3]	2.1 [1.2; 2.5]
	TP second-line	1.3 [1.2; 2.0]	1.1 [0.8; 1.2]	1.4 [0.8; 1.6]	1.6 [1.3; 2.0]	1.9 [1.6; 2.6]
Crea- Clearance [mL·min-^1^·m^−2^]	Control	11 [4; 39]	55 [34; 73]	65 [43; 69]	32 [10; 53]	14 [9; 22]
	TP first-line	0 [0; 8]	60 [41; 83]	41 [21, 68]	55 [25; 55]	4 [0; 36]
	TP second-line	3 [0; 9]	61 [40; 96]	50 [21; 70]	16 [6; 36]	0 [0; 18]
Diuresis [mL·kg^−1^·h^−1^]	Control	0.1 [0.1; 0.4]	0.9 [0.5; 1.6]	0.5 [0.2; 1.0]	0.6 [0.2; 1.2]	0.6 [0.3; 0.9]
	TP first-line	0 [0; 0.1]	1.2 [0.7; 2.4]	0.3 [0; 0.7]	0.4 [0.4; 0.6]	0.1 [0; 0.3]
	TP second-line	0.1 [0; 0.1]	0.8 [0.7; 2.0]	0.3 [0.2; 0.5]	0.4 [0.3; 0.6]	0.2 [0; 0.4]
Bilirubin [mg·dL^−1^]	Control	0.1 [0.1; 0.8]	0.1[0.1; 0.1]	0.1 [0.1; 0.1]	0.1 [0.1; 0.1]	0.1 [0.1; 0.1]
	TP first-line	0.1 [0.1; 0.1]	n.a.	0.1 [0.1; 0.1]	0.1 [0.1; 0.1]	0.1 [0.1; 0.1]
	TP second-line	0.1 [0.1; 0.1]	0.1 [0.1; 0.8]	0.1 [0.1; 0.1]	0.1 [0.1; 0.2]	0.1 [0.1; 0.1]
Protein [g·dL^−1^]	Control	4.1 [3.9; 4.5]	1.9 [1.4; 2.3]	1.5 [1.0; 1.9]	1.1 [0.8; 1.7]	1.4 [1.4; 1.5]
	TP first-line	4.3 [4.1; 5.0]	1.8 [1.5; 2.5]	1.3 [1.1; 1.9]	1.8 [1.0; 1.9]	1.9 [1.3; 2.0]
	TP second-line	4.6 [4.1; 5.1]	1.9 [1.5; 2.3]	1.3 [1.1; 1.8]	1.3 [1.2; 1.8]	1.4 [1.3; 1.8]
Arginine vasopressin [pg·mL^−1^]	Control	239 [195; 291]	8 [5; 12]	11 [3; 96]	9 [4; 19]	9 [8; 14]
	TP first-line	104 [30; 163]	5 [4; 10]	8 [4; 28]	15 [3; 20]	10 [8; 15]
	TP second-line	257 [190; 273]	6 [4; 24]	12 [5; 20]	16 [12; 25]	25 [8; 40]

Values are presented as median [interquartile range].

*Bilirubin, serum bilirubin concentration; Crea-Clearance, creatinine clearance; Creatinine, serum creatinine concentration; Protein, serum protein concentration; TP, Terlipressin*.

All animals developed an increase of liver enzymes over the interventional period. The animals of the TP first-line group showed significantly elevated ASAT and ALAT as compared to the control group (each p < 0.05, see Supplemental Digital Content 5 Fig. 4 and Supplemental Digital Content 6 Fig. 5, ASAT and ALAT). Serum vasopressin levels were lower in the control group as compared to the TP groups (each p < 0.05, see Table 3).

**Figure 5:**
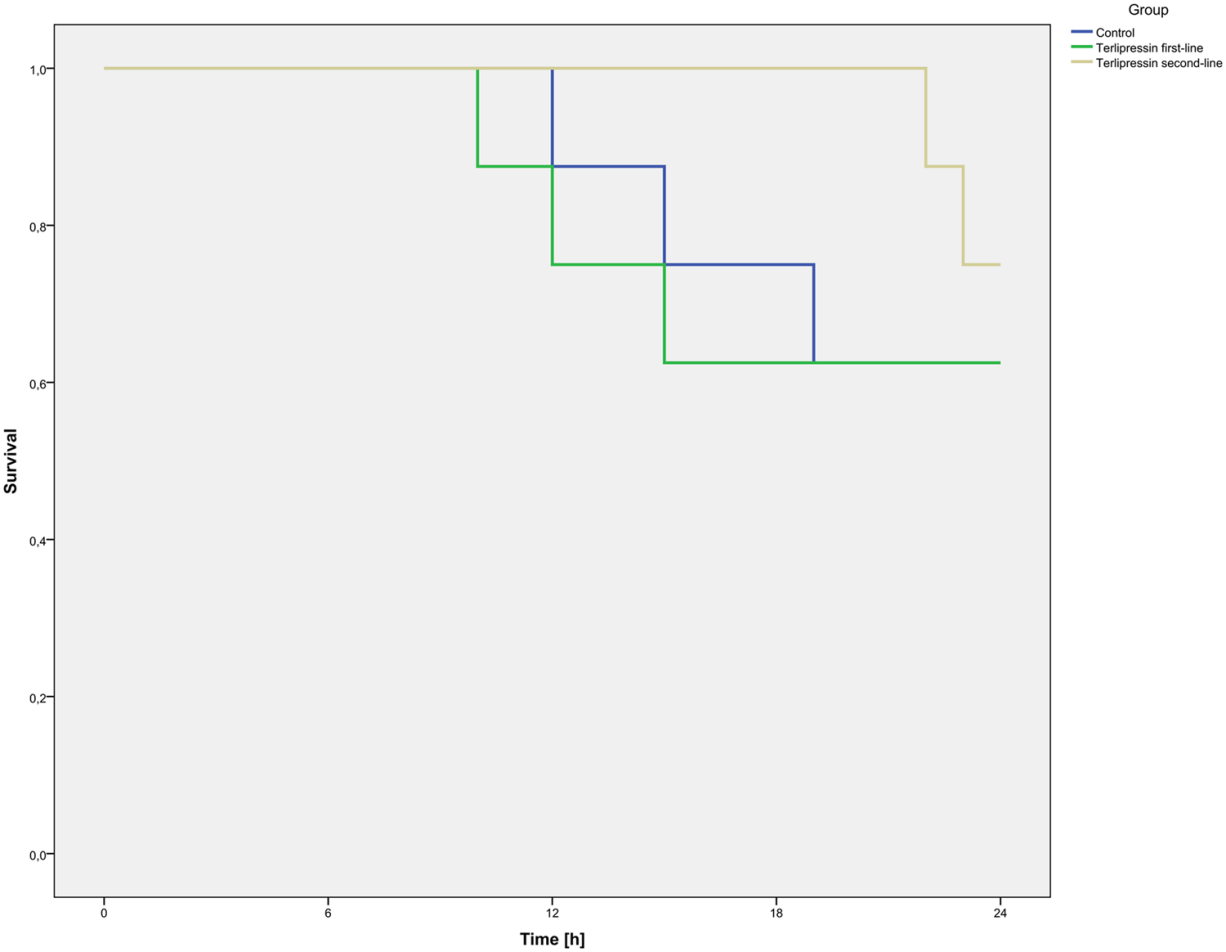
Survival of the study animals. The figure illustrates survival of the study animals over the 24-hour interventional period in a Kaplan-Meyer diagram.

All other measured variables of organ function as well as organ weights and relative organ weights showed no differences between the study groups (see Table 3 and Supplemental Digital Content 7 Table 2, Organ weights).

### Blood cultures

The blood cultures taken at BL were mostly sterile or contained single bacteria of the skin flora, whereas a broad spectrum of intestinal bacteria was detected in the blood cultures taken at shock time. The bacterial load decreased over the interventional period. *Enterococcus faecium* was the most frequently detected bacterial species at the end of the study (see Supplemental Digital Content 8 Figure 6, results from blood cultures).

### Survival

In the control-group and the TP first-line group, each five of eight animals survived the interventional period (62.5%). Six of eight animals survived in the TP second-line group (75%). The mean survival times were 23.6 h (23.1; 24) in the TP second-line group followed by the control-group [20.8 h (17.6; 23.9)] and the TP first-line group [19.6 h, 16.6; 23.6)]. There were no statistically significant differences between the groups regarding 24- h survival (see Fig. 5).

## Discussion

The present study compared the effects of a first-line versus second-line therapy with continuous low-dose TP in ovine septic shock on fluid- and catecholamine requirements as well as organ function and survival. All study animals developed septic shock with hyperlactatemia and acidosis as well as organ dysfunction with the onset of septic shock. Haemodynamics were characterized by a hyperdynamic circulation with high-dose norepinephrine requirements. There were no differences regarding amounts of intravenous fluids or catecholamines and survival between the study groups, though the animals of the TP first-line group tended to receive lower amounts of norepinephrine. No other relevant side effects of terlipressin were detected. Furthermore, no differences between the groups regarding kidney function as measured by diuresis, creatinine or ultrastructural kidney damage quantified by electronic microscopic could be observed.

Septic shock induced in the present study matched the definition of the current international consensus definitions of sepsis and septic shock (Sepsis-3), which define sepsis as a life-threatening organ dysfunction caused by a dysregulated host response following infection^22^. Furthermore, these criteria require not only sepsis with persisting hypotension and the need for vasopressors to maintain a MAP ≥ 65 mmHg but also hyperlactatemia despite adequate volume resuscitation^22^. Infection was induced successfully with peritonitis and consecutive bacteraemia, which was proven by blood cultures (see Supplemental Digital Content 8 Figure 6, results from blood cultures).

A small pilot-trial investigated the effects of first-line AVP versus TP in human septic shock (TERLIVAP). Within this RCT, first-line continuous low-dose administration of TP reduced catecholamine requirements more effectively as compared to AVP and also reduced the risk of new onset tachyarrhythmia^14^. There were no significant differences in norepinephrine requirements between the study groups in the present study, which is an unexpected result that contradicts previous data. One possible explanation might be the dosage of the administered study solutions. Although the applied dose in the study animals (2 µg∙kg^−1^∙h^−1^) was higher than the dosage in the TERLIVAP trial (1.3 µg∙kg^−1^∙h^−1^), which was able to show a significant reduction in catecholamine requirements^14^, underdosing of TP in the present study must be considered as a possible explanation. This is especially true since the required norepinephrine doses were higher than 1 µg∙kg^−1^∙min^−1^ in many animals, suggesting a very severe vasodilatory shock state. The persistent bacteraemia in the present trial might also be interpreted as a sign of severe disease. Since haemodynamics and infection could not be sufficiently stabilized, the present model may be regarded as refractory septic shock. The results from the “Vasopressin and Septic Shock Trial” (VASST) demonstrated beneficial effects of vasopressin administration only in patients with less-severe septic shock^8^, which is another explanation why the therapeutic strategies used in the present trial were ineffective. Other studies using terlipressin in ovine systemic inflammation demonstrated reduction of catecholamine requirements, however, these trials were performed in endotoxemia and not in animals with fulminant abdominal sepsis^11^. In the TERLIVAP trial, significant differences regarding catecholamine requirements between the study groups were measured at least 24 hours after study drug initiation. Thus, another explanation for the lack of differences regarding catecholamine requirements might be the length of the present observational period. Maybe significant differences in catecholamine requirements need some time to occur with continuous infusion of TP, whereas bolus infusion shows immediate hemodynamic effects^23^. It is finally possible, that in severe shock states not only norepinephrine but also non-adrenergic vasopressors need dose adjustment. However, further increase of the terlipressin dose might be associated with increased adverse effects and should therefore be investigated carefully in future trials. There is currently no data available regarding the long-term effects of terlipressin on organ function or adverse effects. Svoboda and colleagues investigated continuous terlipressin administration in catecholamine-refractory septic shock and described no adverse effects^13^. Yildizdas *et al*. used terlipressin as a bolus rescue-therapy in children suffering from septic shock and described no adverse effects or detrimental organ affection as well^24^. Together with the mentioned findings from the TERLIVAP-trial (observational period 48 hours), administration of TP in sepsis seems to be safe in short-term use. However, though the half-life of terlipressin is quite low, one cannot exclude that potential harmful effects on organ function might occur with delay (>48 hours) and were not monitored in the available studies. Accordingly, future trials on terlipressin in sepsis should consider longer observational periods and follow-up of the patients with focus on long-term organ failure.

Furthermore, species related differences in (receptor-)physiology may also play a role in this context^25^. AVP and its synthetic analogues (especially TP) are potent vasopressors, causing vasoconstriction by activation of vasopressin (V)-receptors. While arginine-vasopressin (AVP) has an identical affinity to the (vascular) V_1_ receptor as compared to the (renal) V_2_ receptor (V_1_/V_2_-ratio of 1), terlipressin is more V_1_-selective (V_1_/V_2_-ratio of 2.2) in humans. It should be considered, that this receptor affinity may differ in sheep. Other studies demonstrated beneficial effects of terlipressin in ovine endotoxemia^11^, however, the observed effects did not prove that the V_1_/V_2_-ratio is comparable to human beings.

Increases in haemoglobin and haematocrit levels in septic patients are commonly interpreted as a consequence of both hypovolemia and capillary leakage^26^. The observed increase in haematocrit at shock time indicates that relevant capillary leakage was induced in the present model. Though the cumulative fluid requirements and organ weights were comparable between groups, the haematocrit of the TP second-line group raised significantly over the 24-hour period as compared to the TP first-line group. This might indicate more severe capillary leakage in the TP second-line group, although serum lactate concentrations and catecholamine requirements were comparable between the study groups and offer no hints for a higher severity of septic shock. Additionally, one would expect more fluid requirements in case of higher capillary leakage, however, the cumulative fluid requirements tended to be lower in the TP groups as compared to the control group.

Regarding organ function, there were no differences in acute kidney injury or tubular damage of the animals between the study groups as measured by retention parameters, urinary output and EMTI score. However, there were some differences between the groups in the measured liver enzymes which should be addressed in the following. TP is commonly used in the clinical setting to treat variceal bleeding of the oesophagus. The mechanism behind this is a vasoconstrictive effect of TP on dilated splanchnic blood vessels with consecutive reduction of blood flow and pressure in the portal vein^27^. The increase of liver enzymes in the TP first-line group of the present investigation could be explained by a reduced liver perfusion due to the described mechanism. On the other hand, it has been shown that the reduction in portal venous blood flow by vasopressin agonists is compensated by an increase in hepatic arterial blood flow (so called hepatic artery buffer response)^28,29^. Furthermore, no differences regarding bilirubin levels were observed.

In the TERLIVAP-trial, the investigated septic patients who received continuous low-dose TP showed reduced levels of serum bilirubin as compared to the patients who were treated with norepinephrine or vasopressin. There were no differences between the study patients regarding ASAT, ALAT and activated partial thromboplastin time ratio (aPTTr)^14^. Nevertheless, the observed increase in ASAT and ALAT in the present study appears to be a clear pharmacological effect of TP, since it was most pronounced in the TP first-line group and less pronounced in the second-line group, whereas ASAT and ALAT activities were lowest in the control group. The relevance of this finding should be focussed in future studies.

There are some limitations in the present study which should be addressed:

Since the study was performed in an animal model, results and conclusions should be transferred to clinical settings with caution. Though the hemodynamic pattern of healthy and septic sheep is similar to human beings^30,31^, the effects of vasoactive substances may be different between species, especially regarding the substructure of the vasopressin receptors and the V_1_/V_2_ ratio. Furthermore, though the model is of clinical relevance, one should consider that septic shock was fulminant, and thus any pharmacological intervention may have been futile. It must be noted, that the 24-hour observational period in the present study is quite short for a complex disease like sepsis and for detection of long-term terlipressin effects. Another limitation of the present investigation was the use of HES in septic shock, which was an accepted strategy at the time of initiation of the study with the mentioned maximum dose of 50 ml·kg^−1^ BW^32^. Since fluid therapy was identical among group, this should not induce a relevant bias. Furthermore, the antimicrobial therapy with meropenem failed to eliminate *Enterococcus faecium* (natural resistance against carbapenems) in blood cultures. In future studies using the present model, antimicrobial chemotherapy might include additional gram-positive coverage, e.g. vancomycin.

## Conclusion

In the present study, first-line versus second-line administration of continuous low-dose terlipressin in fulminant ovine septic shock had no influence on norepinephrine and fluid requirements, organ injury or 24-h survival. No beneficial effects of terlipressin were observed, most likely due to the fulminant sepsis with refractory vasoplegia or consecutive underdosing of terlipressin in relation to the severity of the shock state.

## Electronic supplementary material

Supplementary Information
