# Supplementary material for: Comparison of first-line and second-line terlipressin versus sole norepinephrine in fulminant ovine septic shock
Source: Sci Rep. 2018 May 8;8:7105. doi: 10.1038/s41598-018-25570-x (PMC5940827; doi:10.1038/s41598-018-25570-x)
Supplement: Supplementary file 1 — Supplementary Information [file 41598_2018_25570_MOESM1_ESM.pdf]

Supplemental Digital Content

Comparison of first-line and second-line terlipressin versus sole norepinephrine in fulminant ovine septic shock

Tim G Kampmeier[1], Philip H Arnemann[1], Michael Hessler[1], Laura M Seidel[1], Karsten Becker[2], Andrea Morelli[3], Sebastian W Rehberg[4], Christian Ertmer[1]

Variable	BL (n=23)	Shock time (n=23)	p value BL vs shock time
MAP [mmHg]	84 [75; 96]	40 [35; 48]	p<0.001 *
HR [$1 \cdot min^{-1}$]	78 [68; 87]	87 [81; 97]	p<0.001 *
CI [$L \cdot min^{-1} \cdot m^{-2}$]	3.5 [3.1; 3.9]	2.2 [1.9; 2.5]	p<0.001 *
GEDI [$mL \cdot m^{-2}$]	775 [677; 851]	567 [496; 648]	p<0.001 *
CVP [mmHg]	2 [0; 5]	0 [0; 2]	p=0.013 *
SVI [$mL \cdot m^{-2}$]	45 [37; 50]	26 [21; 30]	p<0.001 *
SVV [%]	12 [9; 14]	12 [9; 16]	p=0.768
EVLWI [$mL \cdot kg^{-1}$]	12 [11; 14]	13 [12; 17]	p=0.05
Hb [$g \cdot dL^{-1}$]	7.1 [6.3; 7.8]	10.0 [9.3; 11.6]	p<0.001 *
Lactate [$mmol \cdot L^{-1}$]	0.7 [0.6; 0.9]	1.9 [1.8; 2.0]	p<0.001 *

pH(a) [-lg c(H^+)]	7.44 [7.40; 7.47]	7.43 [7.38; 7.48]	p=0.23
BE [mmol·L^{-1}]	8.0 [5.4, 9.7]	6.0 [2.8; 7.4]	p<0.001 *
DO2I [mL·min^{-1}·m^{-2}]	338 [288; 404]	334 [235; 430]	p=0.617
O2-ER [%]	17 [13; 22]	40 [34; 47]	p<0.001 *
VO2I [mL·min^{-1}·m^{-2}]	60 [46; 72]	126 [97; 167]	p<0.001 *
ScvO2 [%]	83 [80; 89]	62 [56; 68]	p<0.001 *
Creatinine [mg·dL^{-1}]	0.9 [0.8; 1.0]	1.5 [1.2; 1.9]	p<0.001 *
Diuresis [mL·kg^{-1}·h^{-1}]	1.0 [0.7; 3.0]	0.1 [0; 0.1]	p<0.001 *
Crea-Clearance [mL·min^{-1}·m^{-2}]	52 [34; 82]	3 [0; 15]	p<0.001 *
Bilirubin [mg·dL^{-1}]	0.1 [0.1; 0.1]	0.1 [0.1; 0.1]	p=0.958
Temperature [°C]	38.6 [38.3; 38.8]	39.9 [39.4; 40.4]	p<0.001 *

SDC Table 1 **Haemodynamics, variables of oxygen transport, metabolism and organ function at baseline (BL) and shock time**

Data are presented as median [interquartile range]. Wilcoxon signed-rank test was used to compare variables between BL and Shock time; * indicates a statistically significant difference ($p<0.05$).

BE, base excess; BL, healthy baseline measurement; Bilirubin, serum bilirubin concentration; CI, cardiac index; Crea-Clearance, creatinine clearance; Creatinine,

serum creatinine concentration; CVP, central venous pressure; DO_2I, oxygen delivery index; EVLWI, extravascular lung water index; GEDI, global end-diastolic index; Hb, haemoglobin; HR, heart rate; MAP, mean arterial pressure; O_2-ER, oxygen extraction rate; pH(a), arterial potentia hydrogenii; $ScvO_2$, central venous oxygen saturation; SVI, stroke volume index; SVV, stroke volume variation; VO_2I, oxygen consumption index.

SDC Figure 1: Cardiac index

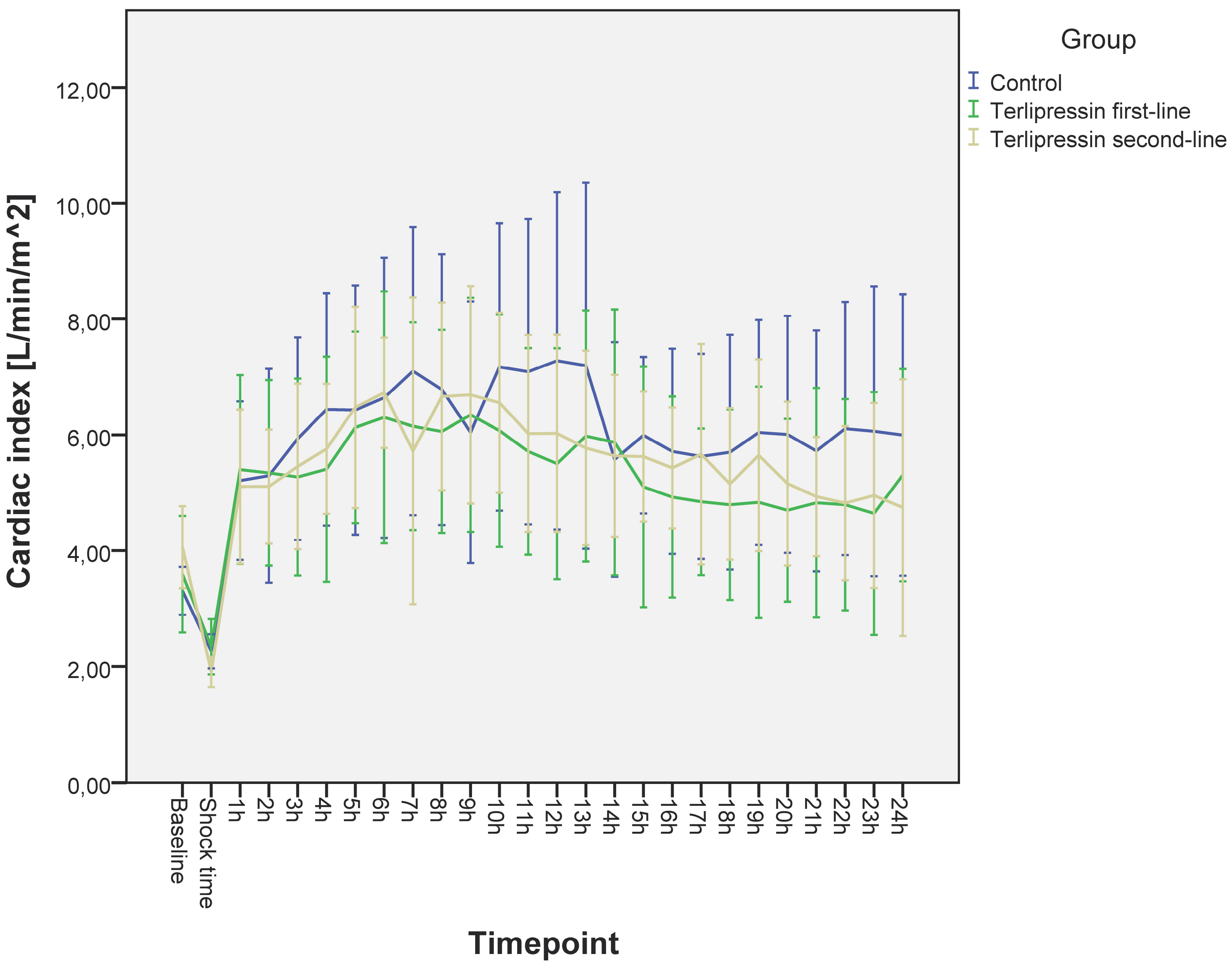

SDC Figure 2: Heart rate

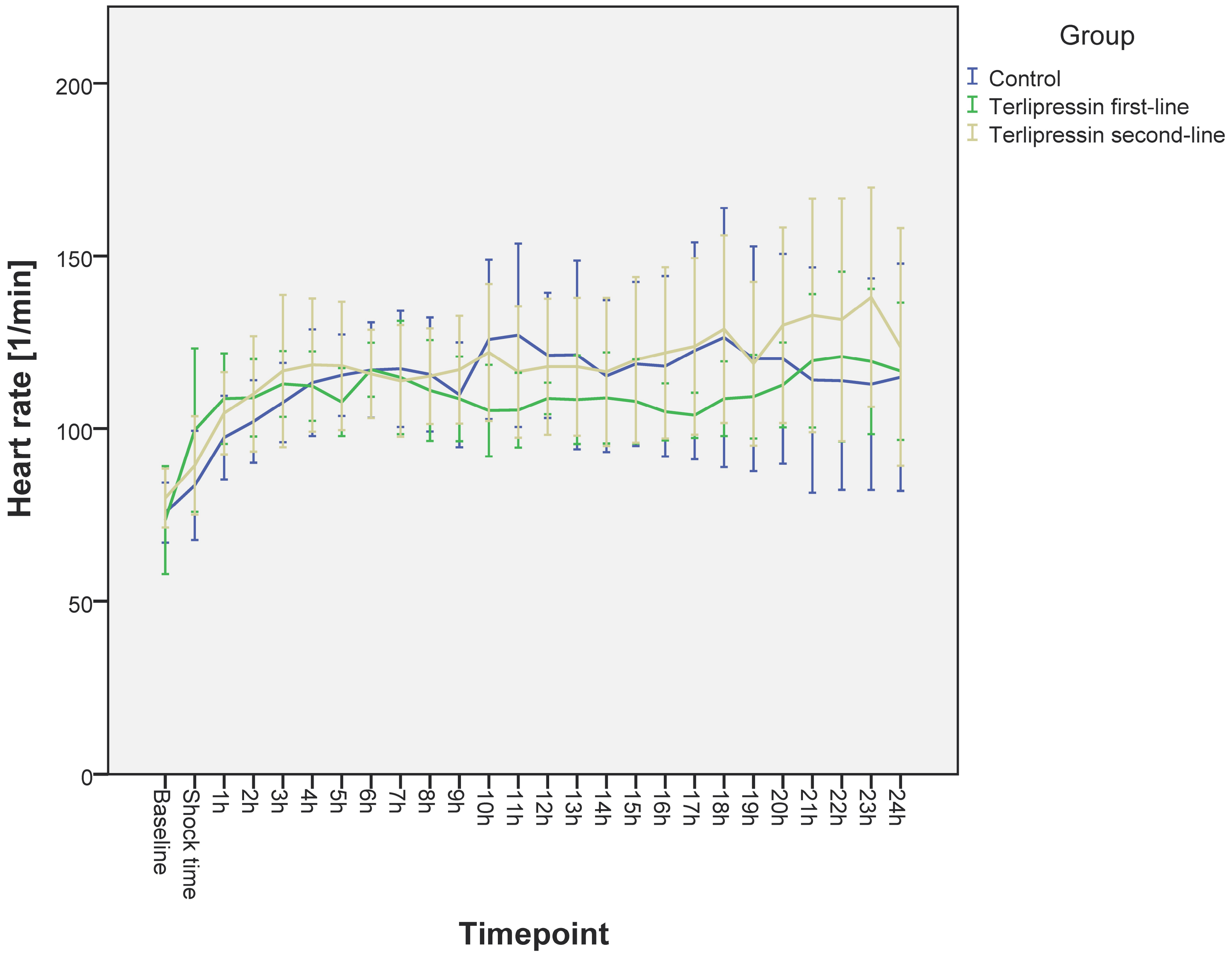

SDC Figure 3: Haematocrit

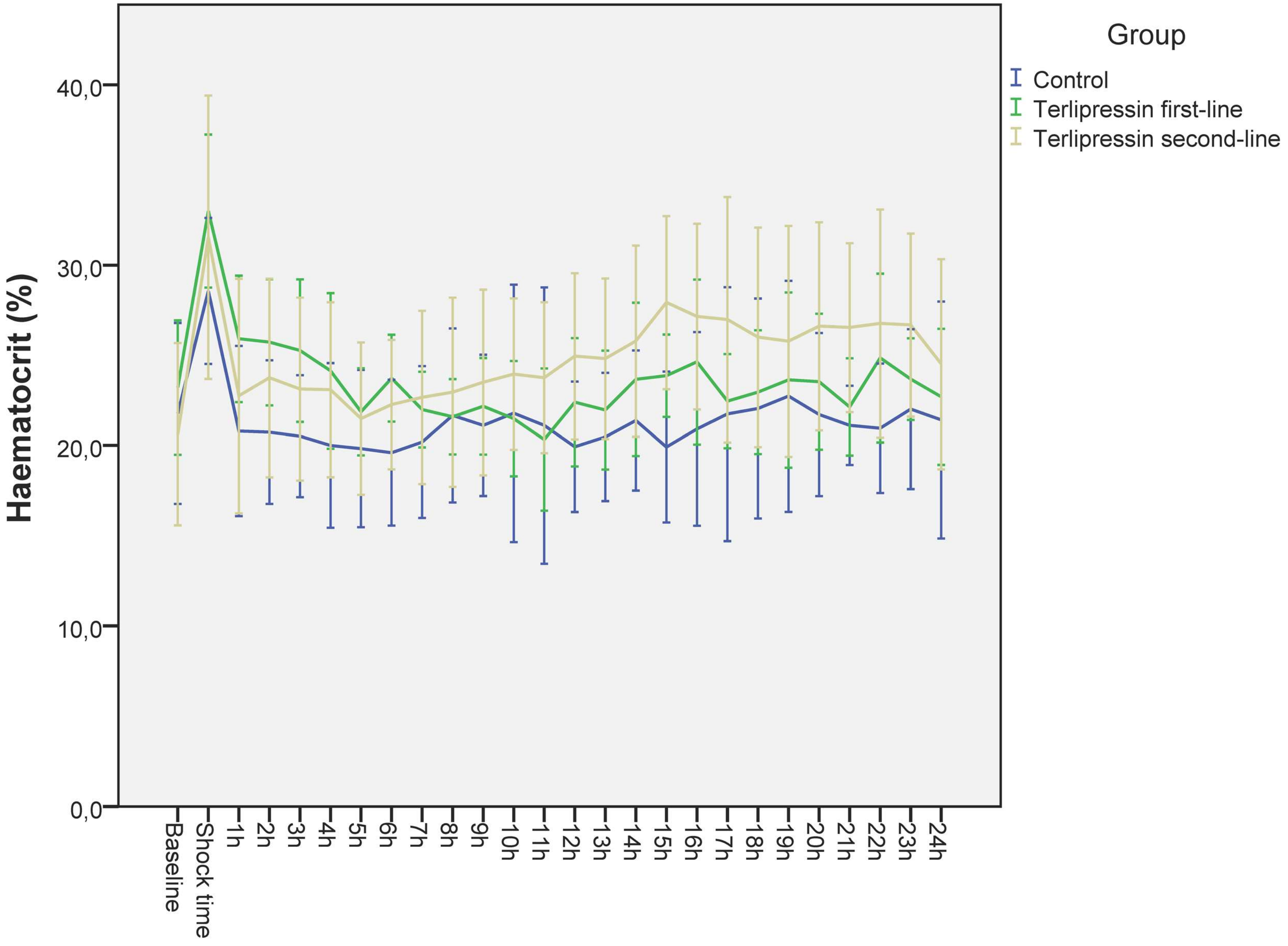

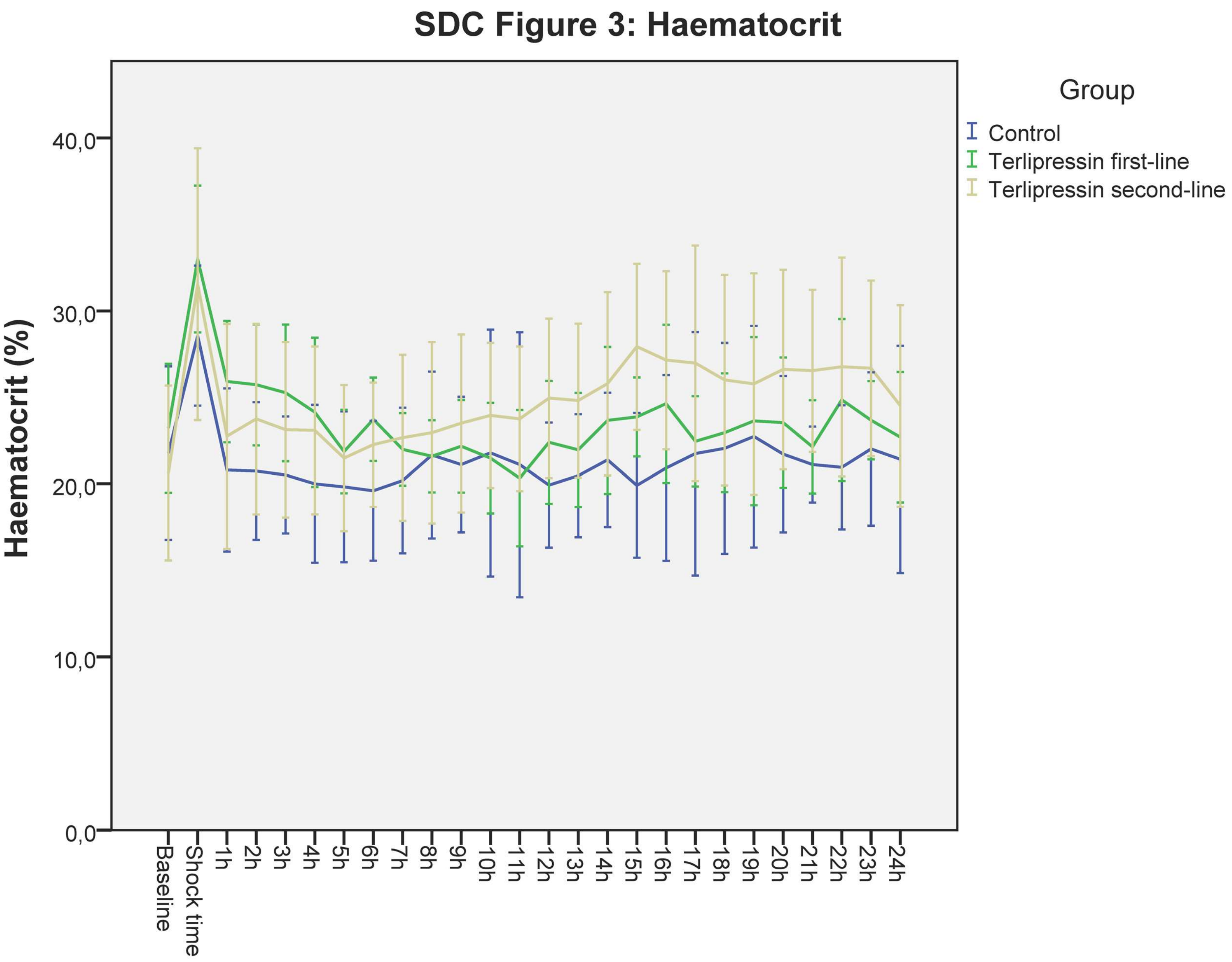

SDC Figure 3: Haematocrit
Haematocrit (%)
40,0
30,0
20,0
10,0
0,0
Timepoint
Baseline
Shock time
1h
2h
3h
4h
5h
6h
7h
8h
9h
10h
11h
12h
13h
14h
15h
16h
17h
18h
19h
20h
21h
22h
23h
24h
Group
Control
Terlipressin first-line
Terlipressin second-line

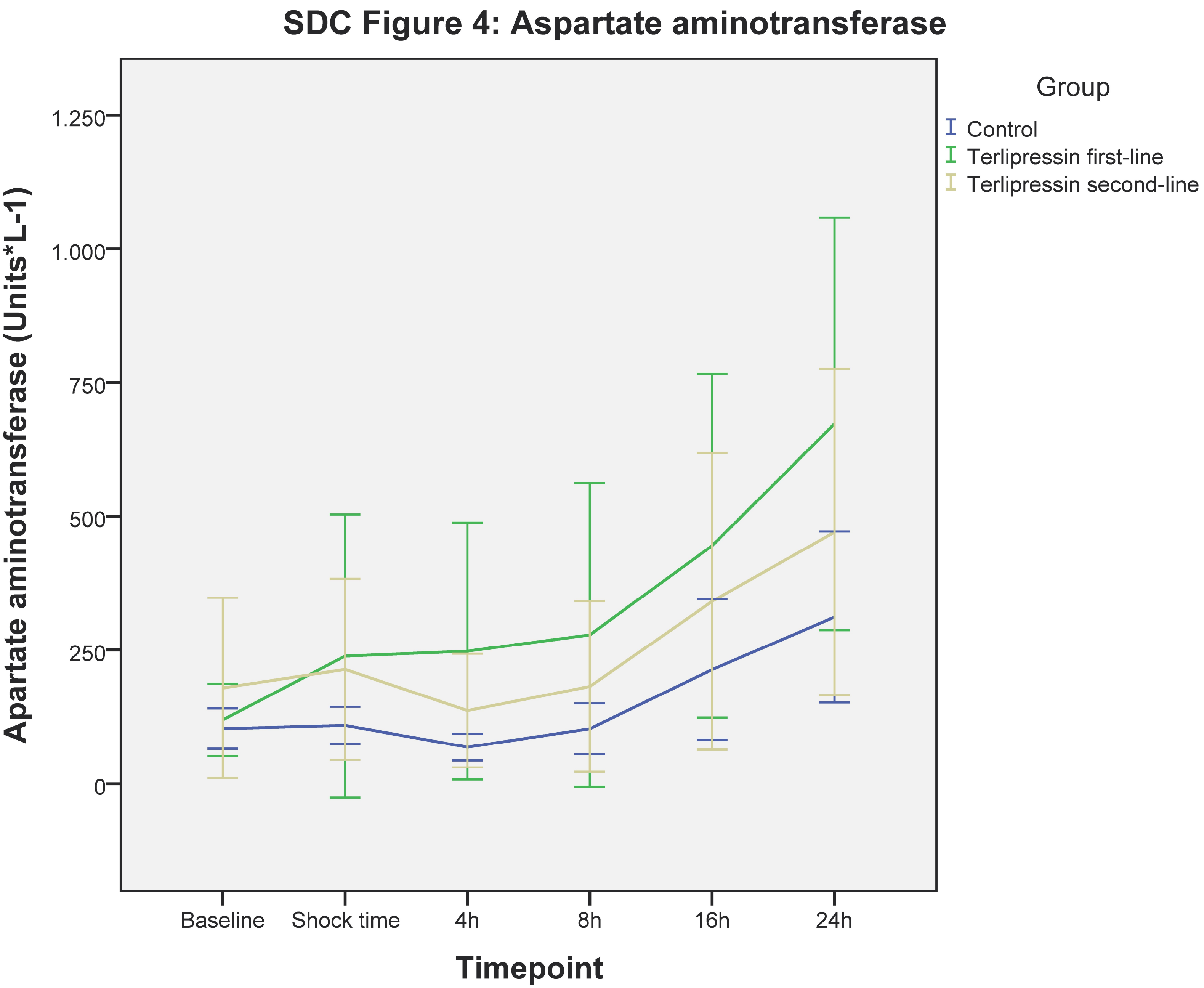
SDC Figure 4: Aspartate aminotransferase
Group
Control
Terlipressin first-line
Terlipressin second-line
Apartate aminotransferase (Units*L-1)
1.250
1.000
750
500
250
0
Baseline
Shock time
4h
8h
16h
24h
Timepoint

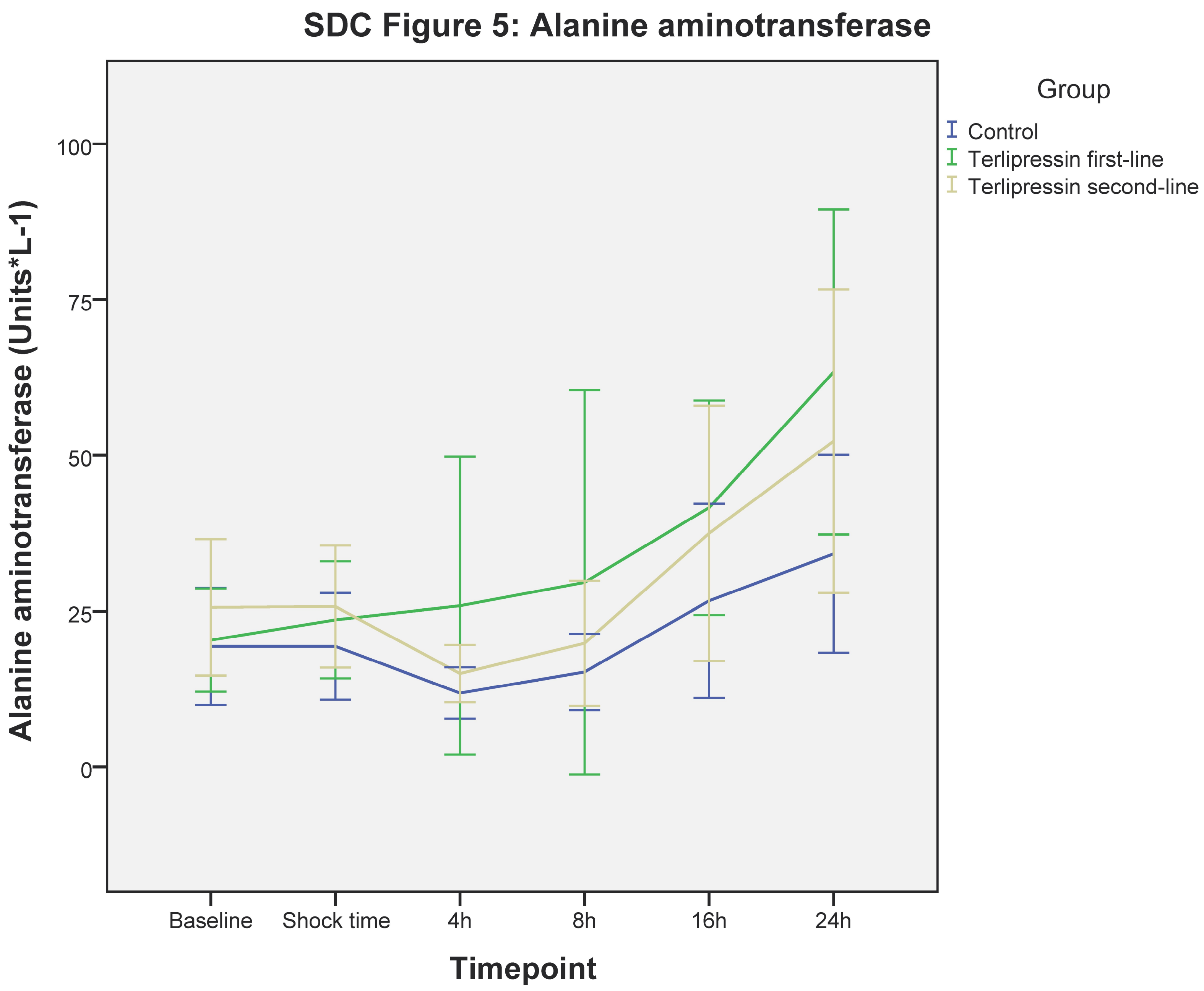

SDC Figure 5: Alanine aminotransferase
Group
Control
Terlipressin first-line
Terlipressin second-line
Alanine aminotransferase (Units*L-1)
100
75
50
25
0
Baseline
Shock time
4h
8h
16h
24h
Timepoint

Variable	Group	Heart	Right kidney	Left kidney	Right lung	Left lung	Ileum
Organ weight [g]	Control	218 [185; 227]	63 [57; 77]	74 [68; 76]	253 [227; 266]	193 [156; 204]	21 [19; 23]
	TP first-line	207 [189; 249]	73 [62; 81]	71 [61; 81]	272 [248; 296]	197 [190; 221]	23 [18; 28]
	TP second-line	204 [175; 218]	72 [64; 78]	73 [60; 82]	263 [222; 291]	186 [155; 227]	19 [17; 22]
Relative organ weight [$g \cdot kg^{-1}$]	Control	5.4 [5.1; 6.4]	1.8 [1.5; 1.8]	1.8 [1.7; 2.0]	6.2 [6.0; 6.8]	4.9 [4.5; 5.3]	0.5 [0.5; 0.7]
	TP first-line	5.2 [4.8; 6.3]	1.8 [1.6; 1.9]	1.8 [1.6; 1.9]	6.8 [5.9; 7.1]	5.1 [4.2; 5.4]	0.6 [0.4; 0.7]
	TP second-line	5.2 [4.7; 6.0]	1.8 [1.5; 2.4]	1.8 [1.5; 2.3]	6.8 [6.3; 7.3]	5.0 [4.7; 5.4]	0.5 [0.4; 0.6]

SDC Table 2: **Organ weights and relative organ weights of the study animals** (as referred to baseline weight)
Values are presented as median [interquartile range]. Kruskal-Wallis test was used for comparison between groups. Post-hoc comparisons were conducted using Dunn's test.

TP, terlipressin

SDC Figure 6: Results from the blood cultures

Baseline		Shock time		8h		16h		24h	
aerob	anaerob	aerob	anaerob	aerob	anaerob	aerob	anaerob	aerob	anaerob
7	7	1	8	n.	n.	n.	n.	n.	n.
10	9	1; 2; 3;	1; 2; 3;	n.	n.				
n.	n.	n.	n.	n.					
7	7	1; 2; 3;	1; 2; 3;	n.	n.	n.	n.		
n.	n.	11	11	12	9	13	13	13	13
9	9	1	n.	13	13	13	13	13	13
n.	n.	1;3;	13; 15; 1	13;	13;				
n.	4;	n.	12;	n.	n.	13;	13; 16;	13; 16;	13;
n.	n.	1;	1;	13;	13;				
n.	n.	1; 11;	1; 11;	n.	17;	n.	n.	13;	13;
18;	n.	1;	1;	n.	n.	13;	13;	13;	13;
n.	n.	2; 3;	2; 3;	13;	n.				
n.	n.	1;	1; 2; 3;	13;	15;	13;	13;	13;	13;
n.	n.	1; 21	1; 21	n.	n.	13;	13;	13;	13;
9;	n.	15;	15;	13;	13;	13;	13;	13;	13;
9;	22;	1; 3;	1; 3;	n.	3;	n.	4; 23		
n.	n.	1; 11; 24	1; 11;	n.	n.	n.	n.	n.	n.
n.	n.	1; 2; 3;	1; 2; 3;	n.	n.	13;	13;	13;	13;
n.	n.	1	1	n.	n.	n.	n.	n.	n.
n.	n.	2;3;	2; 3;	n.	n.	n.	n.	n.	n.
n.	n.	n.	n.	n.	n.	n.	n.	13;	13;
18;	n.	3	11;	n.	n.	n.	n.	n.	n.
n.	n.	3	n.	n.	n.	n.	n.	n.	n.

Nr.	Result
1	Escherichia coli
4	Staphylococcus lentus
5	Mannheimia haemolytica
7	Staphylococcus auricularis
8	Campylobacter spp.
10	Pasteurelle canis
12	Gram-positive rods [not specified]
13	Enterococcus faecium
15	Streptococcus hyointestinalis
16	Stenotrophomonas maltophilia
18	Sphingomonas paucimobilis
19	Gram-positive cocci [not specified]
21	Streptococcus thoraltensis
23	Aerococcus viridans
24	Bacillus species
n .	negative
